# Simple Sequence Repeat and *S*-Locus Genotyping to Assist the Genetic Characterization and Breeding of Polyploid *Prunus* Species, *P. spinosa* and *P. domestica* subsp. *insititia*

**DOI:** 10.1007/s10528-021-10090-7

**Published:** 2021-06-16

**Authors:** Júlia Halász, Noémi Makovics-Zsohár, Ferenc Szőke, Sezai Ercisli, Attila Hegedűs

**Affiliations:** 1 Department of Genetics and Plant Breeding, Szent István University, Ménesi út 44., 1118 Budapest, Hungary; 2 Lövőpetri, Hungary; 3 Department of Horticulture, Faculty of Agriculture, Ataturk University, Erzurum, Turkey

**Keywords:** *Prunus spinosa*, *Prunus domestica* subsp. *insititia*, SSR, Self-incompatibility, *S*-genotype, Polyploid

## Abstract

Polyploid *Prunus spinosa* (2*n* = 4 ×) and *P. domestica* subsp. *insititia* (2*n* = 6 ×) represent enormous genetic potential in Central Europe, which can be exploited in breeding programs. In Hungary, 16 cultivar candidates and a recognized cultivar ‘Zempléni’ were selected from wild-growing populations including ten *P. spinosa*, four *P. domestica* subsp. *insititia* and three *P. spinosa* × *P. domestica* hybrids (2*n* = 5 ×) were also created. Genotyping in eleven simple sequence repeat (SSR) loci and the multiallelic *S*-locus was used to characterize genetic variability and achieve a reliable identification of tested accessions. Nine SSR loci proved to be polymorphic and eight of those were highly informative (PIC values ˃ 0.7). A total of 129 SSR alleles were identified, which means 14.3 average allele number per locus and all accessions but two clones could be discriminated based on unique SSR fingerprints. A total of 23 *S-RNase* alleles were identified and the complete and partial *S*-genotype was determined for 10 and 7 accessions, respectively. The DNA sequence was determined for a total of 17 fragments representing 11 *S-RNase* alleles. ‘Zempléni’ was confirmed to be self-compatible carrying at least one non-functional *S-RNase* allele (*S*_J_). Our results indicate that the *S*-allele pools of wild-growing *P. spinosa* and *P. domestica* subsp. *insititia* are overlapping in Hungary. Phylogenetic and principal component analyses confirmed the high level of diversity and genetic differentiation present within the analysed accessions and indicated putative ancestor–descendant relationships. Our data confirm that *S*-locus genotyping is suitable for diversity studies in polyploid *Prunus* species but non-related accessions sharing common *S*-alleles may distort phylogenetic inferences.

**Supplementary Information:**

The online version contains supplementary material available at 10.1007/s10528-021-10090-7.

## Introduction

*Prunus spinosa* L. (blackthorn or sloe) is a plum species belonging to the *Rosaceae* family (Potter et al. 2007). The diversification of the Eurasian plums dates back to the Oligocene. It is most likely that the extant European lineages of *P. spinosa*, *P. cerasifera* Ehrh. (cherry plum, myrobalan) and *P. domestica* (European plum) descended from an ancestor that migrated from eastern Asia (Chin et al. 2014). *P. spinosa* has a tetraploid genome (2*n* = 4 ×  = 32; Reynders-Aloisi and Grellet 1994). Several diploid species were suspected to contribute to blackthorn genome including *P. cerasifera* and *P. microcarpa* C.A.Mey. (Eryomine 1990; Reynders-Aloisi and Grellet 1994) but a recent phylogenetic analysis suggests that *P. spinosa* has been formed from a diploid ancestor of *P. ramburii* as a result of a polyploidisation event (Reales et al. 2010). It was hypothesized that blackthorn had a key role in the formation of *P. domestica* genome due to an introgressive cross between cherry plum and blackthorn (Crane and Lawrence 1952; Zhebentyayeva et al. 2019); however, it has not yet been confirmed (Horvath et al. 2011).

*P. domestica* subsp. *insititia* L. has been long regarded as a hexaploid (2*n* = 6 ×  = 48) sub-species of *P. domestica* species (Bailey, 1925) and a phylogenetic analysis based on chloroplast DNA sequences supported this notion (Reales et al. 2010). It is a dwarfer and more compact tree with smaller leaves and fruit than other garden plums (Faust and Surányi 1999). Fruits are consumed fresh or processed and the plant is sometimes used as rootstock for garden plum cultivars. Blackthorn is a deciduous wild shrub or small tree, which is self-incompatible and insect-pollinated (Nunes et al. 2006). It propagates vegetatively through root suckers, while its seed is dispersed by mammals and birds (Guitian et al. 1993). Its fruit looks like a small plum, and is often picked in Europe from wild-growing shrubs. It is used to make the alcoholic beverage known as sloe gin but it is also suitable for canning and wine making (Ruiz-Rodríguez et al. 2014). Fruits of *P. spinosa* have great nutritional and functional potential, providing chemical compounds with presumably favourable health-effects (Erturk et al. 2012). The leaves and flowers can be used as tea or source of cosmetic compositions (Sipos and Szabó 2004).

Several companies were established to process different parts of this plant species and their raw material was collected from wild-growing plants. However, it might present problems. The places for collection are usually next to polluted highways and the wild-growing shrubs are not under plant protection, so they are very often infected by fungi (Kovács 2015). Collected fruits differ in quality and hence nowadays there is an increasing demand for the introduction of recognized cultivars with stable and desirable quality properties. Cultivation of commercial cultivars may help overcome such issues and wild genotypes are valuable resources for breeders. Primarily, some interspecific hybrids were bred as rootstocks for plums. Two blackthorn cultivars (‘Nittel’ and ‘Merzig’) were recognized in Germany (Zimmer 2011). However, market demand is varied and national variability can be exploited to satisfy it and hence a blackthorn breeding program has been initiated in Hungary. Main purposes of the project include increased yield, precociousness and bigger fruit size. However, nothing is known about the genetic background of the selected cultivar candidates. Natural hybridization occurs readily between different plum species and in most cases morphological delimitation of the hybrids is impossible (Nielsen and Olrik 2001). Natural hybridization of *P. spinosa* and hexaploid species occurs frequently. The hybrid plants are morphologically similar to *P. spinosa* but are pentaploids (2*n* = 5 ×  = 40) (Halliday and Beadle 1983).

Until now, only few results were reported about the molecular analysis of the less-investigated *P. spinosa*. These papers mainly focused on genetic variability of natural wild populations. The first report highlighted many polymorphisms in the cpDNA of the wild shrub collected from seven deciduous forests across Europe (Mohanty et al. 2000). Later, 16 individual plants from wild populations of Turkey were sampled and subjected to RAPD analysis that proved to be a reliable method to examine genetic relatedness among blackthorn genotypes (Erturk et al. 2009). RAPD has been also efficiently used to evaluate the genetic differentiation of autochthonous blackthorn populations in Germany (Eimert et al. 2016). AFLP analysis was used to estimate the genetic variability of Belgian blackthorn populations and to assess their potential as seed source for gene conservation (Vander Mijnsbrugge et al. 2013) as well as to characterize the genetic differentiation among German, Italian and Hungarian populations influenced by vegetative regeneration and founder effect (Leinemann et al. 2014).

Simple sequence repeats (SSR) or microsatellites have been developed in many *Prunus* species, such as apricot, Japanese plum and cherry (Dirlewanger et al. 2002; Messina et al. 2004; Mnejja et al. 2004) and these markers proved to have high resolution ability to differentiate accessions. In addition, SSR markers developed in one species can be efficiently used in closely related species (Cipriani et al. 1999; Halász et al. 2019; Mnejja et al. 2004; Wünsch 2009). SSRs were also promising to distinguish polyploid *P. cerasus* (Lacis et al. 2011), *P. domestica* and *P. d.* subsp. *insititia* cultivars (Abdallah et al. 2019; Decroocq et al. 2004; Makovics-Zsohár et al. 2017; Urrestarazu et al. 2018) as well as *P. spinosa* (Horvath et al. 2011) genotypes.

*Prunus* species are mainly self-incompatible due to the genetically controlled rejection of self-pollens (De Nettancourt, 2001). This mechanism is commanded by the multiallelic *S*-locus that contains the pistil-expressed *self-incompatibility ribonuclease* (*S-RNase*) and the pollen-expressed *S-haplotype-specific F-box* (*SFB*) genes (Hegedűs et al. 2012; Yamane and Tao 2009). *S*-genotyping of *Prunus* accessions is facilitated by the allele-specific intron length polymorphism (ILP) shown by both *S-RNase* introns (Wiersma et al. 2001; Sonneveld et al. 2003; Sutherland et al. 2004). Three *S-RNase* and *SFB* alleles were sequenced in *P. cerasifera* (*S*_3_, *S*_9_ and *S*_10_) and *P. domestica* (*S*_5_, *S*_6_ and *S*_9_) by Sutherland et al. (2008). Recently, additional *S*-alleles have been identified and hexaploid plum cultivars have been *S*-genotyped (Makovics-Zsohár et al. 2016; Abdallah et al. 2019). The analysis of *S*-locus in *P. spinosa* focused primarily on the pollen-specificities and 37 *SFB* alleles were sequenced together with 7 *S-RNase* alleles (Nunes et al. 2006; Vieira et al. 2008).

The polyploid plum species grow wild and readily form interspecific hybrids in Hungary, a secondary centre of diversification, and such populations may provide genotypes selected for specific breeding purposes. The aim of this study was to estimate the genetic polymorphism of perspective blackthorn and plum genotypes selected in a Hungarian breeding program, to determine and characterize their *S-RNase* alleles and assess the overlap of *S*-allele pools among different plum species. We also wanted to evaluate the efficiency of SSR and *S*-locus markers in the genetic analysis of polyploid plum species.

## Materials and Methods

### Plant Material

A total of 16 polyploid plum (nine *Prunus spinosa* L., four *P. domestica* subsp. *insititia* L. and three *P. spinosa* × *P. domestica*) cultivar candidates and a newly released blackthorn cultivar (ʻZempléni’) originated from different geographic locations (Supplementary Fig. 1) in the Carpathian basin (Hungary and Slovakia, Central Europe) were analysed in this study (Table 1). Scion wood was collected from the wild-growing perspective plants and grafted onto Myrobalan rootstocks. The trees were grown in an experimental orchard at Lövőpetri, Hungary, with 5 m × 3 m spacing. All sampled plants were approx. 7 years old.

**Table 1:** Taxonomic classification, place of origin, *S*-genotype, important characteristics and suggested utilization of polyploid plum cultivar candidates analysed in this study

Genotypes	Species	Place of origin (village, county, country)^a^	*S*-genotype^b^	Important characteristics and utilization^c^
A1	*P. domestica* subsp. *insititia*	Apagy, SSB, HUN	***S***_**A**_*S*_B_*S*_G_*S*_U_	AW 21 mm, fresh consumption, spirit, jam
B3	*P. domestica* subsp. *insititia*	Berkesz, SSB, HUN	***S***_**C**_*S*_I_***S***_**J**_*S*_K_*S*_L_	AW 35 mm, rootstock
D2	*P. spinosa*	Szeghalom, BEK, HUN	*S*_3-1_***S***_**B**_*S*_I_***S***_**M**_	AW 16 mm, high yield, spirit, jam, syrup, cosmetics
D4	*P. spinosa*	Mezőberény, BEK, HUN	***S***_**12**_*S*_C_***S***_**K**_*S*_N_	AW 17 mm, no spines, spirit, jam, syrup, cosmetics
D5	*P. spinosa*	Mezőberény, BEK, HUN	***S***_**3-1**_*S*_M_*S*_O_***S***_**P**_	AW 17 mm, densely spiny, spirit, jam, syrup, cosmetics
L1	*P. spinosa* × *P. domestica* (controlled pollination)	Lövőpetri, SSB, HUN	*S*_3-1_***S***_**B**_***S***_**C**_***S***_**M**_	AW 21 mm, freestone, fresh consumption, canned fruit, spirit
L2	*P. spinosa* × *P. domestica *(controlled pollination)	Lövőpetri, SSB, HUN	*S*_12_*S*_B_*S*_C_*S*_K_*S*_T_	AW 20 mm, susceptible to cracking, spirit
L4/1	*P. spinosa* × *P. domestica* (controlled pollination)	Lövőpetri, SSB, HUN	*S*_H_*S*_M_*S*_P_*S*_X_	AW 14 mm, spirit, jam, syrup
S3	*P. spinosa*	Sárospatak, SSB, HUN	*S*_C_*S*_P_	AW 18 mm, spirit, syrup, jam
L5	*P. spinosa*	Lövőpetri, SSB, HUN	*S*_12_*S*_B_*S*_H_*S*_M_	AW 20 mm, high yield, fresh consumption, canned fruit, spirit
S3/B	*P. spinosa*	Sárospatak, SSB, HUN	*S*_H_*S*_K_	AW 17 mm, spirit, syrup, jam
U1	*P. spinosa*	Újkenéz, SSB, HUN	*S*_A_*S*_P_*S*_X_*S*_V_	AW 16 mm, late ripening time, small canopy, spirit, syrup, jam
T1	*P. domestica* subsp. *insititia*	Mezőladány, BAZ, HUN	***S***_**3-1**_*S*_12_*S*_C_***S***_**D**_***S***_**U**_*S*_Y_	AW 22 mm, freestone, high yield, fresh consumption, spirit, canned fruit
T4	*P. domestica* subsp. *insititia*	Mezőladány, BAZ, HUN	*S*_3-1_*S*_12_*S*_C_*S*_D_*S*_U_*S*_Y_	AW 21 mm, similar to T1 but fruits are softer and flatter, fresh consumption, spirit, canned fruit
ZE	*P. spinosa*	Nagykapos, NAM, SLO	*S*_12_*S*_C_*S*_J_*S*_Q_	AW 23 mm, self-compatibility, fresh consumption, spirit, syrup, jam
Z3	*P. spinosa*	Pácin, BAZ, HUN	***S***_**B**_***S***_**D**_*S*_E_*S*_V_	AW 17 mm, spirit, syrup, jam
S2	*P. spinosa*	Sárospatak, SSB, HUN	*S*_C_*S*_L_	AW 15 mm, freestone, food supplement, syrup, spirit

^a^*ZE* ʻZempléni’ cultivar, *BAZ* Borsod-Abaúj-Zemplén county, *BEK* Békés county, *NAM* Nagymihályi county, *SSB* Szabolcs-Szatmár-Bereg county, *HUN* Hungary and *SLO* Slovakia

^b^Alleles labelled by bold symbols have been sequenced

^c^AW Average fruit width

### Pollination Test

Self-compatibility of ‘Zempléni’ cultivar was tested in the field in three subsequent years, 2016–2018, according to Nunes et al. (2006). Before anthesis, approximately 100 flower buds were bagged to exclude bees. Fruit development was continuously monitored, and the percentage of fruit set was recorded after two months.

### DNA Extraction and PCR Conditions

Genomic DNA was extracted from fully expanded young leaves using a DNeasy Plant Mini Kit (Qiagen, Hilden, Germany). DNA concentrations and purification parameters were measured using a Nanodrop ND-1000 Spectrophotometer (Thermo Fisher Scientific, Waltham, MA, USA). For microsatellite analysis, a set of 11 SSR primer pairs were selected on the basis of previous reports on different *Prunus* species: CPSCT018, CPSCT 021 (Mnejja et al. 2004), CPDCT044 (Mnejja et al. 2005), BPPCT007, BPPCT025, BPPCT037, BPPCT038, BPPCT039 and BPPCT040 (Dirlewanger et al. 2002), EPDCU 5100 (Howad et al. 2005) and ASSR63 (Xie et al. 2006). The forward primers were labelled with 6-FAM fluorescent dye for detection in a capillary genetic analyzer. For *S*-genotype analysis, PCRs were performed using the consensus primer pairs of PaConsI-F/PaConsIR2 (the forward primer was fluorescently labelled with 6-FAM) amplifying the first intron of *Prunus S-RNase* gene, and PaConsII-F/PaConsII-R to amplify the second intron, according to the protocol described for the primers (Sonneveld et al. 2003, 2006).

PCR reactions were carried out in a PTC 200 thermocycler (MJ Research, Budapest, Hungary) using the program described for the primers. Approximately 20–80 ng of genomic DNA was used for PCR amplification in a 25 μl reaction volume containing 10 × Dream*Taq*™ Green buffer (Fermentas, Szeged, Hungary) as well as KCl and (NH_4_)_2_SO_4_ at a ratio optimized for robust performance of Dream*Taq*™ DNA Polymerase in PCR with final concentrations of 4.5 mM MgCl_2_, 0.2 mM of dNTPs, 0.2 μM of the adequate primers and 0.75 U of Dream*Taq*™ DNA polymerase (Fermentas).

### Cloning and Sequencing

PCR products were ligated to the TA cloning vector pTZ57R/T (InsTAclone PCR Cloning Kit, Thermo Scientific) and transformed into DH5α competent *Escherichia coli* cells. Positive transformants were identified by blue/white selection according to the protocol. Plasmid DNA was isolated with the EZ-10 Spin Column Plasmid DNA kit (Bio Basic Inc., Markham, Canada) and sequenced using an ABI 3500 XL Genetic Analyzer (Applied Biosystems, Foster City. CA, USA). For each fragment, the nucleotide sequences of two clones were determined in both directions with sequencing primer. Homology searches of DNA sequences were carried out using blastn at NCBI and aligned and presented with MEGA5.1 (Tamura et al. 2011) and BioEdit v. 7.2.0. (Hall 1999), respectively.

### Allele Sizing and Data Analysis

To check the PCR amplifications and determine the approximate sizes of the SSR and *S-RNase* first intron alleles, 4 µl of the PCR products were separated by electrophoresis in 1.2% TAE agarose gels for 2 h at 100 V and DNA bands were visualized by ethidium bromide staining. The *S-RNase* second intron PCR products were separated in 1.5% TAE agarose gels for 2 h at 120 V, and DNA bands were also visualized by ethidium bromide staining. Fragment lengths were estimated by comparison with the GeneRuler 1 kb DNA Ladder (Thermo Fisher Scientific). To determine the exact size of the SSR alleles and *S*-*RNase* first intron region, the fluorescently labelled PCR products were run on an automated sequencer ABI PRISM 3100 Genetic Analyzer (Applied Biosystems). Peak Scanner 1.0 software and the GS500 LIZ size standard (Applied Biosystems) were used for determination of size in bp. The polymorphic information content (PIC) of markers was calculated according to Hildebrand et al. (1992).

The evolutionary divergence between sequences was estimated using the Poisson correction model (Zuckerkandl and Pauling 1965). The analysis involved 22 amino acid sequences. All positions containing gaps and missing data were eliminated. There was a total of 123 positions in the final dataset. Evolutionary analyses were conducted in MEGA5.1.

For the phylogenetic analysis, two datasets were used. First, each detected allele from SSR was used separately and then it was completed with *S-RNase* second intron genotyping data. Alleles were scored as present (1) or absent (0). The unweighted pair-group average algorithm (UPGMA) was used to construct a dendrogram based on Dice similarity coefficients with the software PAST 2.17c (Hammer et al. 2001). Numbers on major branches represent bootstrap supports from 2000 replicates. Principal component analysis (PCA) was also carried out using PAST software.

## Results and Discussion

### SSR Marker Analysis

In recent years, many studies have dealt with SSR marker development and fingerprinting of *Prunus* species. In this study, a total of 11 SSR loci were screened using primers designed for different *Prunus* species and tested for cross-amplification within the *Prunus* genus (Dirlewanger et al. 2002; Howad et al. 2005; Mnejja et al. 2004, 2005). Loci were chosen based on the detected polymorphism level. Amplification was not successful in some samples in case of CPSCT018 while ASSR63 was monomorphic amplifying a single fragment of 157 bp in each of the samples. Therefore, data of the latter two primers were excluded from the evaluation. The remaining 9 loci proved to be polymorphic (Table 2). The genotyping data for each of the 9 SSR markers presented in Supplementary Table 1 are readily available. In each genotype, 1–6 alleles were detected according to the different ploidy levels of accessions. As expected, the presumable tetraploid, pentaploid and hexaploid genotypes amplified maximum 4, 5 and 6 alleles in each locus, respectively. In cases when the number of detected alleles was lower than the expected number, we hypothesized the presence of null alleles or that one or two of the alleles were present in more copies.

**Table 2:** SSR loci analysed in the polyploid *Prunus* accessions, locus type, linkage group of their localization, species of origin, reference, annealing temperature, allele size range, number of alleles and unique alleles detected, number of differentiated genotypes and polymorphic information content (PIC) values

Primer name	Locus type	Linkage group	Species	Reference	Ta (°C)	Allele size range	Number of alleles	Number of unique alleles	Number of detected genotypes	PIC
BPPCT007	SSR	G3	peach	Dirlewanger et al. (2002)	57	122–154	17	5	16	0.91
BPPCT025	SSR	G6	peach	Dirlewanger et al. (2002)	57	132–208	24	7	16	0.94
BPPCT037	SSR	G5	peach	Dirlewanger et al. (2002)	57	100–120	5	2	6	0.54
BPPCT038	SSR	G5	peach	Dirlewanger et al. (2002)	57	110–140	6	0	8	0.77
BPPCT039	SSR	G3	peach	Dirlewanger et al. (2002)	57	122–146	7	0	11	0.76
BPPCT040	SSR	G4	peach	Dirlewanger et al. (2002)	57	120–154	12	1	15	0.89
CPDCT044	SSR	G2	almond	Mnejja et al. (2005)	58	162–252	24	11	16	0.94
CPSCT021	SSR	G2	plum	Mnejja et al. (2004)	56	124–208	26	9	16	0.95
EPDCU5100	EST-SSR	G1	almond	Howad et al. (2005)	57	124–144	8	1	11	0.83
PaConsII	*S-RNase*	G6	sweet cherry	Sonneveld et al. (2003)	58	~ 490– ~ 3000	23	6	16	0.93

SSR genotyping of polyploids might be challenging (Mason 2015). However, the tested primers provided clear amplification and reliable allele identification was not even hindered by stuttering (representative chromatograms are shown in Supplementary Fig. 2). A total of 129 different SSR alleles were identified, which means 14.3 average allele number per locus ranging from 5 (BPPCT037) to 26 (CPSCT021). Seven of the markers used in this study were also included in genotyping 53 hexaploid *P. domestica* accessions and amplified a similar number of alleles. The biggest difference in amplified allele number was seen in case of BPPCT039 that produced many more alleles in the hexaploid samples (Makovics-Zsohár et al. 2017; Urrestarazu et al. 2018). Horvath et al. (2011) also used the BPPCT007 primers and found 19 alleles (122–160 bp) in 14 blackthorn genotypes. Those numbers are very similar to the ones obtained in the present study (17 alleles, 122–154 bp). They detected 32 alleles in the BPPCT025 loci, which exceeds the allele number (25) in this locus of the analysed Hungarian accessions. The CPSCT021 marker was analysed in 8 Japanese plums (Mnejja et al. 2004) and in function of the smaller sample set, the detected number of alleles (4) and size range was smaller than in this study. Primers for the CPDCT044 almond locus worked properly also in Japanese plums and amplified a total of 7 alleles in eight almond cultivars (Mnejja et al. 2005), while 3.4-times more alleles were identified in the tested 17 polyploid *Prunus* genotypes presumably due to their enhanced ploidy level.

Among 11 SSR markers, BPPCT037 proved to be the most informative primer in *P. lannesiana* and amplified five-times more alleles than in this study (Kato et al. 2011). In the tetraploid *P. cerasus* it amplified 16 alleles, which exceeded the number of amplified alleles for other loci including BPPCT007, BPPCT038, BPPCT039 and BPPCT040 (Antonius et al. 2012). Considering that those latter primer pairs amplified almost identical (BPCCT038 and BPPCT039) or larger numbers of alleles (BPPCT007 and BPPCT040) in polyploid plums and sour cherry, the limited performance of BPPCT037 is not explained by the smaller number of samples or differences in ploidy level. It indicates that levels of allelic variability in SSR loci can vary species by species and BPPCT037 is more polymorphic in *Prunus* species of the Cerasus subgenus compared to those in Prunophora. The most alleles were amplified by CPSCT021 in our study while this marker was less polymorphic in diploid *Prunus* species (Mnejja et al. 2010).

The EPDCU5100 locus amplified only two to four alleles in cherry, peach, apricot and almond (Mnejja et al. 2010; Halász et al. 2019); however, in this study we detected a higher number (8) of alleles. Interestingly, Zhang et al. (2014) also detected 8 alleles in the EPDCU5100 locus by analysing 94 Chinese apricot accessions. Although this marker is characterized by lower polymorphism compared to others used in this study (e.g. CPSCT021 or BPPCT025), it amplifies enough alleles to be efficiently used in a diverse sample set like polyploid *Prunus* accessions.

Allele sizes varied from 100 to 252 bp. The highest size range was caught in the CPDCT044 locus (a difference of 90 bp), while the lowest range was shown in BPPCT037 and EPDCU5100 (20 bp in both cases). Since wide discrepancies were detected in the number of amplified alleles, the polymorphic information content of loci changed accordingly, ranging from 0.54 (BPPCT037) to 0.95 (CPSCT021). The average PIC value was 0.84. This is much higher than PIC values of SSR markers in other tetraploid species including cotton and coffee (Lacape et al. 2007; Moncada and McCouch 2004) and slightly higher than 0.73 for *P. serotina* (Guzmán et al. 2018) and identical to the value obtained for sour cherry (Lacis et al. 2011). A PIC value exceeding 0.7 is considered to be highly informative (Hildebrand et al. 1992) indicating that 8 of the applied 9 SSR markers used in this study have great potential for molecular studies in polyploid *Prunus* species. The lowest PIC value was also higher than 0.44 that is considered to be the threshold for moderately informative markers.

The CPDCT044 locus had the most unique alleles (11), while loci with limited allele numbers and PIC values (BPPCT038 and BPPCT039) had not produced alleles occurring exclusively in a given genotype. It is interesting to realize that the least informative locus, BPPCT037, had amplified two alleles exclusively occurring in a single genotype, one of those in S2 and another in ʻZempléni’ (Supplementary Table 1).

The genotypes carrying a maximum of 4 alleles in the analysed loci, are likely to be tetraploids (D2, D4, D5, L5, S2, S3, S3/B, U1, Z3 and ʻZempléni’), while a genotype characterized by 5 alleles in at least one locus (e.g. L1 in BPPCT007) is consistent with the notion that it is a pentaploid hybrid of *P. spinosa* × *P. domestica*. The fact that L2 or L4/1 amplified only 1–4 alleles in each locus cannot rule out its supposed pentaploid genome as they are artificial hybrids of a tetraploid *P. spinosa* and hexaploid *P. domestica*. We also had four *P. domestica* subsp. *insititia* accessions with a presumable hexaploid genome structure. However, only 1–5 alleles were amplified in three of the four accessions and only B3 had six alleles in the CPPCT044 locus. The same range of alleles was amplified in hexaploid *P. domestica* subsp. *insititia* by Wünsch (2009) indicating that ploidy level cannot be assessed based on SSR data since the absence of alleles (due to deletion of the corresponding genomic regions) or their presence in multiple copies may not allow the determination of the copy number of alleles. Therefore, cytological or flow cytometry analyses are needed to unequivocally determine ploidy levels (Halász et al. 2011).

The polymorphism detected in the tested loci is in agreement with the resolution power of the markers since all SSR primers characterized by PIC values higher than 0.90 were able to discriminate 16 genotypes of the 17 accessions tested. One pair of *P. domestica* subsp. *insititia* accessions (T1 and T4) produced identical genotypes by using all 9 markers and could be identified as putative clones (see later). It means that using a single marker out of the four markers with higher than 0.9 PIC values allowed the discrimination of the polyploid *Prunus* accessions tested in this study. Similarly, a combination of only two markers with lower PIC values, BPPCT039 and EPDCU5100 might be enough to differentiate polyploid *Prunus* genotypes. Such high levels of genetic variability are common in case of self-incompatible, generatively propagated species (Kodad et al. 2013), and useful markers were identified for the genetic analysis of polyploid plums.

### *S*-allele Profiling

*P. spinosa* is a self-incompatible (SI) species, and its sexual incompatibility is governed by the highly polymorphic *S*-locus (Nunes et al. 2006). Since under a functional SI system, homozygosity is impossible in the *S*-locus, this polymorphism might be also exploited for the estimation of genetic variation. Until now, only two works dealt with the analysis of *S*-locus in wild blackthorn individuals (Nunes et al. 2006; Vieira et al. 2008). However, *P. domestica* has both self-incompatible and self-compatible cultivars (Kota-Dombrovska and Lacis 2013) and its hexaploidy genome further increases the allelic diversity at the *S*-locus. This is the first study to determine the *S*-allele constitution of Central European blackthorn genotypes and to test its application for genetic studies.

**Fig. 1:**
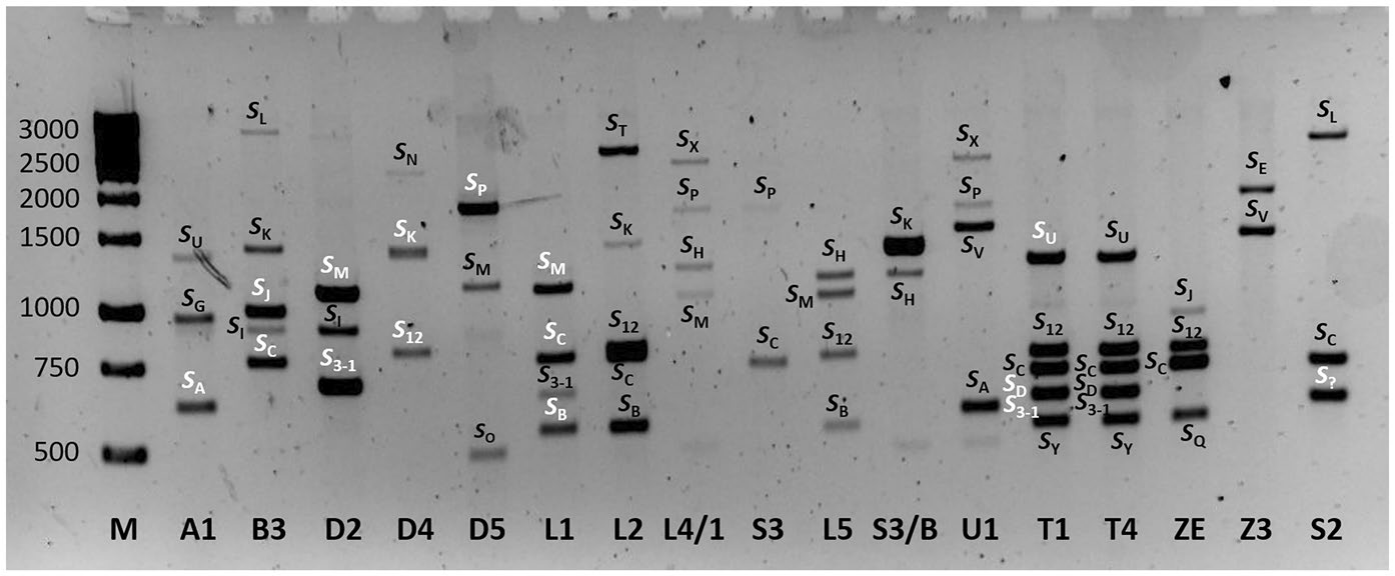
Polymerase chain reaction (PCR) analysis of the second intron region of *self-incompatibility ribonuclease* (*S-RNase*) alleles in 17 polyploid *Prunus* accessions using the PaConsII primer pair. Lane M, 1-kb + ladder; accessions are identified with their labels as mentioned in Table 1. The alleles determined by DNA sequencing are indicated by white characters. S? refers to an allele which requires further study to identified

For *S*-allele profiling in *Prunus* genus, the ILP technique based on the pistil-expressed *S-RNase* gene is the most common method (Sonneveld et al. 2003). We used the PaConsI-F/PaConsIR2 (Sonneveld et al. 2003, 2006) and PaConsII-F/PaConsII-R (Sonneveld et al. 2003) primer pairs designed to amplify the first and second intron region of the gene, respectively. The amplification of first intron region did not produce fragments of acceptable intensity in case of A1, D4, L2, S2 and S3/B samples and resulted in only partial amplification for the others with 1 to 4 fragments of putative *S*-alleles. A total of 36 fragments were amplified for 17 accessions, an average of 2.1 alleles per accession. The PaConsII primer pair proved to give the most informative banding pattern with 68 fragments across the 17 accessions resulting in an average of 4 alleles per genotype. Amplified fragment sizes ranged from 490 bp to ~ 3000 bp (Fig. 1, Table 2). The statistics reflecting the diversity in *S*-locus and BPPCT025 SSR locus are in good agreement. These loci are in proximity on the *Prunus* linkage group 6; therefore, similar allelic variation is expected. The different fragment lengths were assigned to self-incompatibility alleles, provisionally labelled by letters (*S*_A_ − *S*_Y_).

To check the amplified fragments indeed code for *S-RNase* alleles, some PCR products of the tetraploid *P. spinosa* D2, D4, D5 and Z3, the pentaploid *P. spinosa* × *P. domestica* hybrid L1 and the hexaploid *P. domestica* subsp. *insititia* A1, B3 and T1 were cloned and sequenced. Although the size variations of the second intron regions occurring within the range of 2,500 bp length allowed the reliable identification for most of the alleles, there were pairs of alleles with almost identical fragment sizes (e.g. *S*_3-1_ and *S*_D_). DNA sequencing was also required to discriminate such alleles since some genotypes (T1 and T4) carried both alleles while only a single band could be observed on gels (Fig. 1). It also explains why the allele represented by an approx. 700-bp fragment in S2 accession is yet to be identified.

The DNA sequence was determined for a total of 17 fragments representing 11 *S-RNase* alleles of 8 accessions. The sequences were deposited in the DDBJ/EMBL/GenBank databases under the following accession numbers, MN052897–MN052899 and MN069629–MN069642. Homology searches undoubtedly declared that all fragments were homologous to the *Prunus* self-incompatibility ribonuclease gene (Supplementary Table 2).

**Fig. 2:**
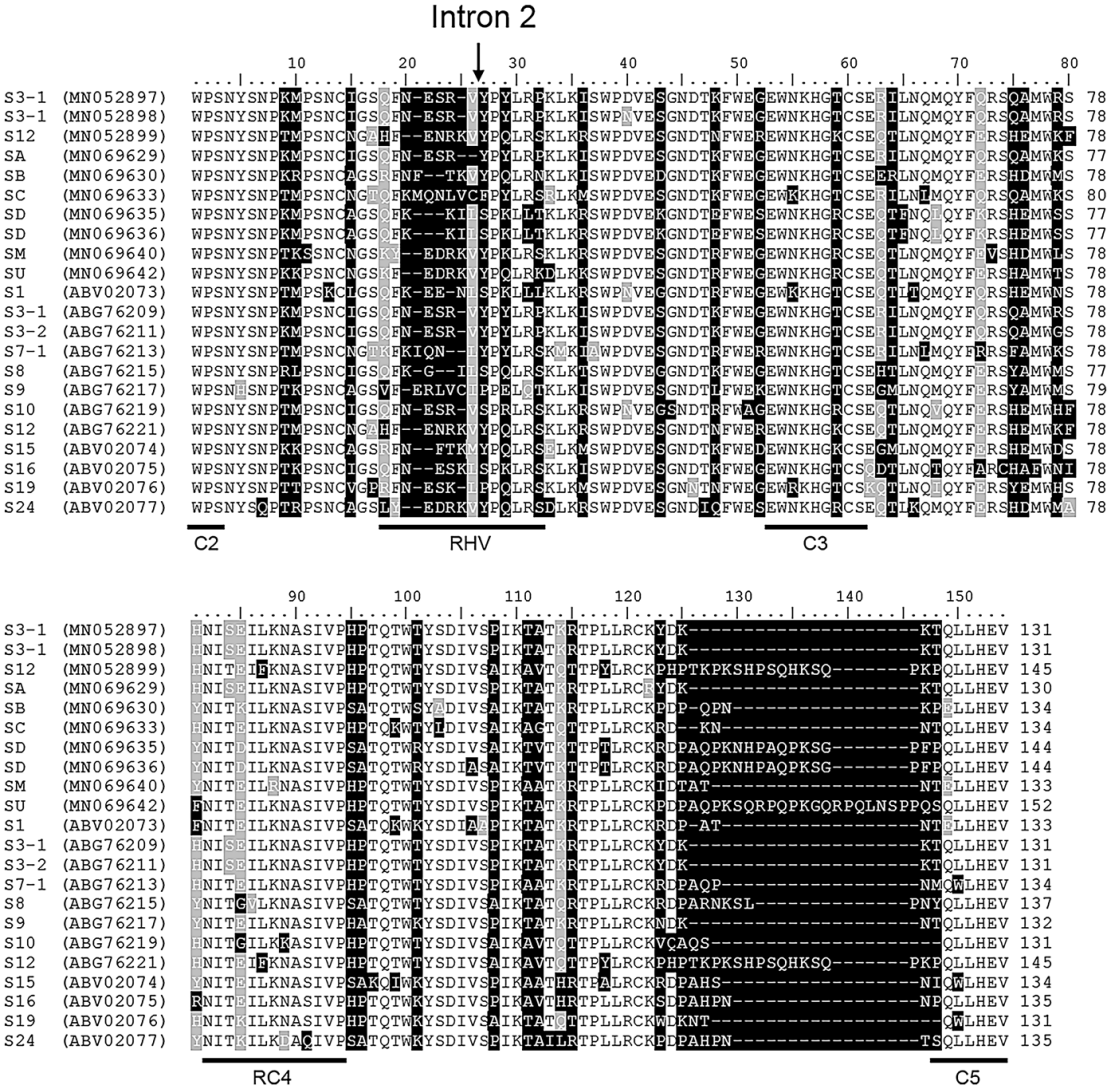
Multiple alignment of 10 deduced amino acid sequences of the polyploid *Prunus S*-RNase alleles identified in this study and 12 *Prunus spinosa S*-RNase sequences available in GenBank database. The conserved regions (C2–C5) and the hypervariable region (RHV) are underlined according to Ushijima et al. (1998). The arrow indicates the position of the second intron

The alignment of the deduced amino acids from C2 to C5 of the 10 polyploid *Prunus S*-*RNase* alleles identified in this study and 12 *P. spinosa S*-*RNase* sequences available in database (determined by Nunes et al. 2006 and Vieira et al. 2007) is shown in Fig. 2. The conserved regions and the RHV are shown according to Ushijima et al. (1998) and the position of the *S-RNase* second intron found within the RHV region is also indicated. Variable amino acid positions and conserved amino acid replacements are marked by black and grey background colours, respectively. The identified sequences show the characteristic structural motifs of *Prunus S*-RNase enzymes with C2–C5 conserved regions and two highly variable motifs (including the RHV and the region between RC4 and C5). The blastn analysis, the alignment and the estimates of divergence between sequences (Supplementary Table 3) indicated that both T1 and D5 carry an *S-RNase*-allele identical to that described by Nunes et al. (2006) as *S*_3-1_. Another allele in D4 proved to be identical to *S*_12_ described by the same authors in Portuguese wild-growing *P. spinosa* populations.

We have cloned and sequenced certain *S-RNase* alleles from different genotypes (Supplementary Table 2). D2, L1 and Z3 had the *S*_B_-allele, all of which are identical to each other. One copy of *S*_C_ and *S*_M_ sequences was slightly shorter ending just before the C5 region; and hence those sequences were not included in the alignment. However, the two copies of the allele *S*_3-1_ (identified in T1 and D5 genotypes), and *S*_D_ (carried by T1 and Z3) showed minor polymorphisms. The *S*_3-1_ sequences differed in two and four bases in the intron and exon regions of the gene, respectively; that resulted in a single non-synonymous amino acid replacement (D → N) in the third exon. The two sequenced *S*_D_-alleles differed in only one base resulting in a non-synonymous amino acid replacement (V → A) between the RC4 and C5 regions. Considering the positions of such intra-allelic variations in the alleles *S*_3-1_ and *S*_D_ are outside the most variable regions of the gene that have a confirmed role in determining allele-specificity (Matton et al. 1997; Ortega et al. 2006; Ushijima et al. 1998), they are not expected to change allele specificity, although controlled pollination would be required to clarify it.

The sequence analysis of *S*_J_-allele cloned from B3 revealed the presence of a premature stop codon (AAA → TAA) in the last amino acid position of the RHV region. Consequently, the transcript of the gene is supposed to encode a truncated protein lacking a large 3’ segment of the third exon of *S-RNase* gene including the His amino acid within the C3 conserved region that is thought to be involved in enzyme catalysis (Broothaerts et al. 1995). Hence, *S*_J_-RNase is supposed to be non-functional. *S*_J_ is also carried by cultivar ʻZempléni’ that has been characterized by autogamous fruit set ratios in isolated flowers ranging from 12 to 26% over three years, confirming this cultivar to be self-compatible (SC). Artificial field pollination experiments have not revealed self-compatible (SC) *P. spinosa* genotypes in Portugal (Nunes et al. 2006) but polyploid *Prunus* species (e.g. tetraploid sour cherry) generally include both self-compatible and self-incompatible genotypes (Hauck et al. 2006; Hegedűs et al. 2012), and hence the occurrence of SC blackthorn genotypes cannot be ruled out. ʻZempléni’ is the first blackthorn cultivar that had received state recognition in Hungary, and self-compatibility would be a preferred character in cultivation. However, further analysis (e.g. sequencing the pollen *SFB* alleles) is required to clarify the molecular basis of SC in ʻZempléni’; since self-compatibility in sour cherry, another allotetraploid species is induced by the accumulation of at least two non-functional *S*-haplotypes (Hauck et al. 2006).

A total of 23 *S-RNase* alleles were identified in 17 tested polyploid *Prunus* genotypes with six unique alleles found only in a single genotype. In conclusion, the PIC value of PaConsII primer pair reached the values characteristic for the most efficient SSR markers. Only two *P. domestica* subsp. *insititia* accessions (T1 and T4) shared a common *S*-genotype. They also had identical SSR profiles in all tested loci. Both accessions derived from the same region but far enough (4 km apart) to exclude that vegetatively spreading runners had been sampled. In addition, they presented slight phenotypic variations presumably derived from bud sport mutations occurred during clonal propagation. Grafting has been long used in countryside to propagate perspective individuals. It is important to remark that such accessions cannot fertilize each other when planted in a common orchard.

The *S*-genotypes are consistent with the taxonomic classification of the studied accessions. As in case of SSR analysis, the three presumed ploidy levels (4 × , 5 × and 6 ×) resulted in different allele numbers in the analysed accessions. The largest *S*-allele number (6) was detected in T1 and T4 *P. domestica* subsp. *insititia* accessions. The smallest allele number (2) was recorded in S3 and S3/B genotypes. Ten accessions had four *S*-alleles (A1, D2, D4, D5, L1, L4/1, L5, U1, Z3 and ʻZempléni’ cultivar) (Table 1). Two genotypes (B3 and L2) carried 5 alleles. B3 is a presumable hexaploid *P. domestica* subsp. *insititia* and L2 is a putative pentaploid hybrid arisen from a controlled cross of *P. spinosa* and *P. domestica*. The detection of fewer *S-RNase* alleles than expected can be explained by several reasons including the preferential amplification of specific alleles in consensus PCR (Walsh et al. 1992; Kota-Dombrovska and Lacis 2013), the matching sizes of amplicons representing different alleles (Kodad et al. 2008), the presence of null alleles or the multiple appearance of a given allele in the *S*-locus (Pairon et al. 2008; Tsukamoto et al. 2010). However, the presence of multiple copies of an *S*-allele is only possible in case of non-functional alleles. Moncada and McCouch (2004) measured a significant drop in the allele numbers and PIC values of the markers used for fingerprinting cultivated tetraploid coffee compared to the diploid *Coffea* species. It was explained by the facts that diploids are outcrossing and come from diverse geographical origins, while the tetraploids are predominantly self-pollinating, and all originated from Ethiopia. The high allele numbers and PIC values in the studied polyploid *Prunus* germplasm are consistent with the notion of self-incompatibility for most accessions. The great number of *S*-alleles in a self-incompatible species is normal since the number of *S*-alleles often exceeds 40, like in case of almond and apple (Kodad et al. 2008; Halász et al. 2011). In addition, the allo-polyploid genome structure in itself promotes the emergence of new alleles (Ainouche et al. 2014).

*S*_C_ was the most frequent *S-RNase* allele carried by 9 accessions, each *S*_12_ and *S*_B_ was carried by 6, and *S*_3-1_ by 5 accessions (Table 3). Interestingly, both *S*_3-1_ and *S*_12_-alleles were also found in three of the 11 tested Portuguese wild-growing blackthorn individuals (Nunes et al. 2006). It indicates these alleles are quite frequent in blackthorn accessions growing in distant geographic positions. From 23 *S-RNase* alleles, ten and two alleles were specific to tetraploid *P. spinosa* and hexaploid *P. domestica*, respectively, while 11 were carried by both species. It indicates that the *S*-allele pools of *P. spinosa* and *P. domestica* subsp. *insititia* are overlapping in Hungary. The *S*_T_-allele occurred only in a *P. spinosa* × *P. domestica* hybrid and hence its origin remains unknown. Currently, only three *P. domestica S-RNase* allele sequences are known (Sutherland et al. 2008), all of which seem to be different from *S*_G_ and *S*_U_ based on the length of second intron regions. Sequencing and a more detailed analysis of *S*-alleles in hexaploid European plum will be required to clarify those alleles.

**Table 3:** Approximate and precise allele sizes (bp) based on the estimation from agarose gel pattern of the PCR fragments amplified using the PaConsII primers (Sonneveld et al. 2003) and DNA sequencing, respectively, allele frequencies and occurrences in the tested species

Allele	Size (bp)	Frequency	Occurrences^a^			
			*Ps*	*Pi*	*Ps* × *Pd*	Total
*S*_3-1_	713	6.33	2	2	1	5
*S*_12_	815	7.59	3	2	1	6
*S*_A_	627	2.53	1	1	0	2
*S*_B_	559	7.59	3	1	2	6
*S*_C_	783	11.39	4	3	2	9
*S*_D_	716	3.80	1	2	0	3
*S*_E_	~ 2100	1.27	1	0	0	1
*S*_G_	~ 980	1.27	0	1	0	1
*S*_H_	~ 1250	3.80	2	0	1	3
*S*_I_	~ 950	2.53	1	1	0	2
*S*_J_	1013	2.53	1	1	0	2
*S*_K_	~ 1450	5.06	2	1	1	4
*S*_L_	~ 3000	2.53	1	1	0	2
*S*_M_	1113	6.33	3	0	2	5
*S*_N_	~ 2500	1.27	1	0	0	1
*S*_O_	~ 490	1.27	1	0	0	1
*S*_P_	~ 1800	5.06	3	0	1	4
*S*_Q_	~ 610	1.27	1	0	0	1
*S*_T_	~ 2700	1.27	0	0	1	1
*S*_U_	~ 1400	3.80	1	2	0	3
*S*_V_	~ 1500	2.53	2	0	0	2
*S*_X_	~ 2600	2.53	1	0	1	2
*S*_Y_	~ 590	2.53	0	2	0	2

^a^*Ps Prunus spinosa*, *Pi P. domestica* subsp. *insititia*, *Ps* × *Pd P. spinosa* × *P. domestica* hybrids

In cultivated self-incompatible species, it is a crucial point to check sexual compatibility because it determines reliable fruit set. Cultivars carrying different *S*-alleles (belonging to different cross-incompatibility groups) must be inter-planted in orchards; therefore, the information of *S*-genotypes is necessary for both growing and breeding (Yamane and Tao 2009). Using the ILP marker strategy, complete or partial *S*-genotypes of cultivar candidates were determined (Table 1). Altogether 10 complete *S*-genotypes were identified, and only two accessions (putative clones) shared identical alleles (T1 and T4). Since all other cultivar candidates had different *S*-allele compositions, it makes them potential pollen donors in any combinations. However, polyploidy may limit pollen fertility. Similarly to *P. cerasus*, blackthorn is considered to have equal chance for all possible chromosome pairing during meiosis (Vieira et al. 2008). In such a case, when two tetraploid accessions sharing two *S*-alleles (e.g. D2 × D5 or D4 × ‘Zempléni’) are crossed, less than 20% of the pollen grains (those carrying no matching alleles with the plant to be pollinated) will be able to grow in the pistil to the ovary. It may seriously limit the amount of compatible pollen grains and decrease fruit set.

### Estimation of Genetic Distances

**Fig. 3:**
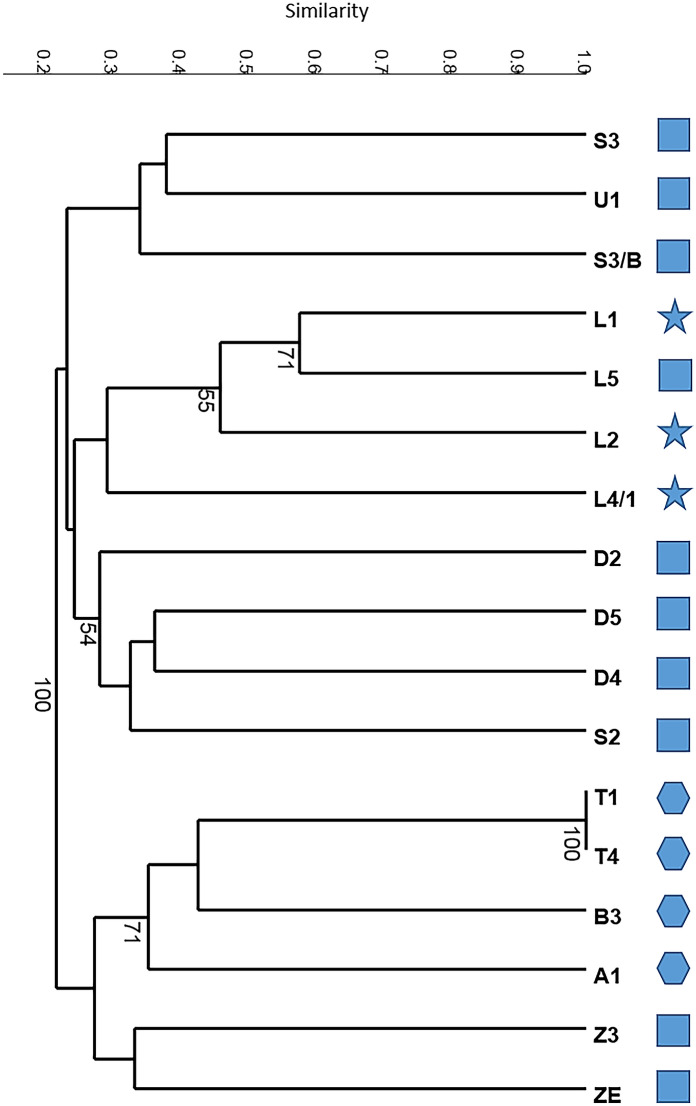
Unweighted pair-group average (UPGMA) dendrogram based on Dice indices among SSR genotypes in 9 loci of 17 native *Prunus spinosa* (squares), *P. domestica* subsp. *insititia* (asterisks) and *P. spinosa* × *P. domestica* hybrid (hexagons) accessions. Numbers indicate bootstrap values (percentage of 2000 replicates). Bootstrap values greater than 50% are shown

Altogether 429 alleles amplified in nine SSR loci of 17 cultivar candidates were used to estimate the genetic distance and perform UPGMA cluster analysis based on Dice indices (Fig. 3). The cophenetic correlation value of the dendrogram was 0.81, indicating a quite good fit that was statistically significant at the 1% level according to Lapointe and Legendre (1992). The dendrogram grouped the accessions into four sub-clusters, two of which contained only *P. spinosa* accessions, another group consisted of *P. spinosa* and its hybrids while the fourth clade grouped *P. domestica* subsp. *insititia* and *P. spinosa* accessions. A similar co-clustering of some *P. spinosa* and *P. domestica* subsp. *insititia* genotypes was also observed using sequence-based genotyping (Zhebentyayeva et al. 2019).

All *P. domestica* subsp. *insititia* genotypes grouped together (A1, B3, T1 and T4) with strong bootstrap support. T1 and T4 were identical. Two interspecific (*P. spinosa* × *P. domestica*) hybrids (L1 and L2) were close to each other and formed a common clade with a *P. spinosa* genotype (L5), which received strong bootstrap support. Another hybrid, L4/1 was also positioned in this clade. The phylogenetic analysis suggests that L5 was a progenitor genotype for the studied hybrids. Such a relationship is further supported by the *S*-genotypes, since L5 shared two *S*-alleles with each of the three hybrids (L1, L2 and L4/1).

The cluster analysis was also carried out on the merged dataset of SSR and *S*-genotype markers by incorporating the distribution pattern of 68 detected *S-RNase* fragments among the 17 tested accessions. The basic structure of the dendrogram remained unchanged; however, the position of some accessions changed considerably (Supplementary Fig. 3). For example, ʻZempléni’ cultivar and Z3 formed a sister clade to the *P. domestica* subsp. *insititia* clade in SSR genotyping, while the inclusion of *S*-genotypes placed ʻZempléni’ cultivar within this clade together with hexaploid accessions. This is obviously not correct and presents a warning signal that *S*-locus information might not be useful for phylogenetic analysis. ʻZempléni’ shares two *S*-alleles with B3 (*S*_C_ and *S*_J_) and with T1-T4 (*S*_12_*S*_C_). Since ʻZempléni’ is *P. spinosa* while the rest are *P. domestica* subsp. *insititia*, matching *S*-alleles must be the consequence of chance effects rather than phylogenetic relationships. In loquat, pairs of cultivars (e.g. ʻMogi’ and ʻAlcácer’, ʻWanzhong’ and ‘Mc Beth’) with identical *S*-genotypes were confirmed to be distantly related (Gisbert et al. 2009). Similar results were obtained for sweet cherry (Wünsch and Hormaza 2002) and almond (Halász et al. 2019) cultivars.

**Fig. 4:**
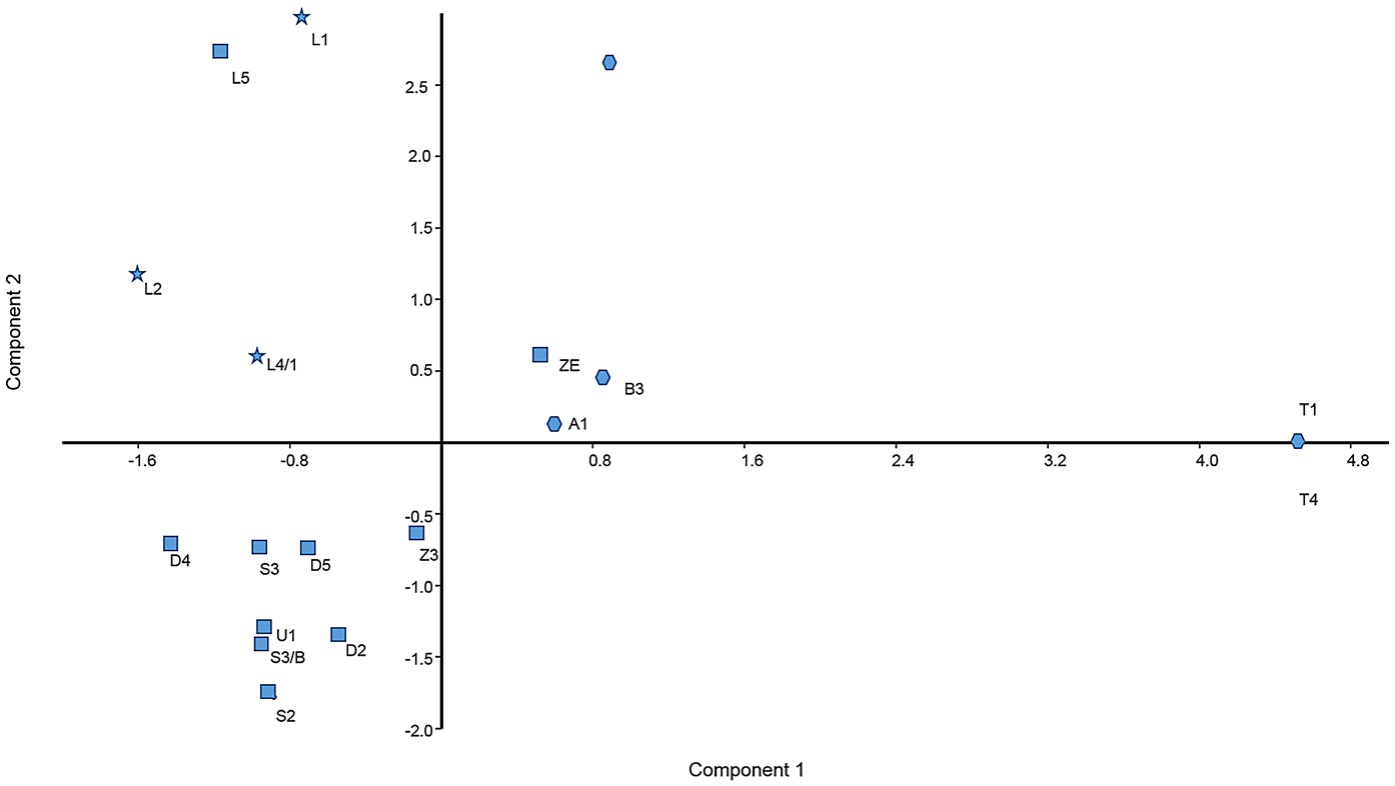
Distribution of *Prunus spinosa*, *P. domestica* subsp. *insititia* and *P. spinosa* × *P. domestica* cultivar candidates on the two first principal component analysis axes determined from SSR genotyping

PCA analysis confirmed the information provided by the dendrogram and supplied further details. The first two principal axes accounted for 18.8% and 10.2% of the total variation. PCA analysis confirmed that the hexaploid T1 and T4 genotypes were identical in SSR genotypes and it revealed their great genetic distance from the rest of the tested accessions (Fig. 4). The two additional *P. domestica* subsp. *insititia* A1 and B3 accessions are also placed close together and into a different position from *P. spinosa*. Interestingly, PC2 could only slightly differentiate among two pairs of hexaploid plums (T1-T4 and A1-B3). The first two principal components sharply separated the *P. spinosa* × *P. domestica* hybrids and their presumable progenitor *P. spinosa* accession, L5, from the rest of the studied genotypes. PCA was efficiently used for plum diversity studies based on both phenotyping and genotyping (Horvath et al. 2011; Yilmaz et al. 2009). The phylogenetic analysis and PCA plot confirmed a high level of diversity and genetic differentiation present within the analysed genotypes and supported putative ancestor–descendant relationships for some accessions.

## Conclusions

High genetic diversity was observed among 17 polyploid plum accessions (*P. spinosa*, *P. domestica* subsp. *insititia* and *P. spinosa* × *P. domestica* hybrid cultivar candidates) in Hungary using both SSR and *S*-locus based genotyping. Eight of the applied 9 SSR markers used in this study had great potential for molecular studies in polyploid *Prunus* species with CPSCT021 being the most polymorphic. The multiallelic *S*-locus also provided considerable variability that can be exploited for molecular fingerprinting and diversity studies (it is important for breeding and cultivation); however, phylogenetic analysis may be confused by not related accessions sharing common *S*-alleles. A total of 23 *S-RNase* alleles were identified in 17 tested polyploid *Prunus* accessions, partial DNA sequences were determined for 11 of those. The cultivar ‘Zempléni’ was found to be self-compatible and one of its *S-RNase* alleles (*S*_J_) was determined to contain a premature stop codon and hence it is suggested to be non-functional. It makes the tetraploid *P. spinosa* an important plant species to test general validity of the ‘one-allele match’ model of self-compatibility in sour cherry described by Hauck et al. (2006). Comparing the results from the phylogenetic clustering and PCA analysis, the taxonomic status of the accessions could be confirmed, and progenitor genotypes could be proposed in some cases. Most of these genotypes were selected from wild populations, which means they represent part of the genetic variations of wild-growing Hungarian blackthorn and *P. domestica* subsp. *insititia* species. It confirms that Hungary has rich genetic potential for the exploitation and valorisation of currently under-utilized polyploid plum species.

## Supplementary Information

Below is the link to the electronic supplementary material.

Supplementary Figure 1: The geographic origin of the analysed Central European *Prunus spinosa, P. domestica* subsp. insititia and *P. spinosa* × *P. domestica* accessions (TIF 536 KB)

Supplementary Figure 2: Unweighted pair-group average (UPGMA) dendrogram based on Dice indices among SSR in 9 loci and S-genotypes of 17 native *Prunus spinosa* (squares), *P. domestica* subsp. insititia (asterisks) and *P. spinosa* × *P. domestica* hybrid (hexagons) accessions. Numbers indicate bootstrap values (percentage of 2000 replicates). Bootstrap values greater than 50% are shown (TIF 638 KB)

Supplementary Figure 3: Representative Peak Scanner traces for the CPSCT021 microsatellite alleles amplified with 6-FAM-labelled primers in ten polyploid *Prunus* accessions and GS500 LIZ size standard. Coordinates on the X-axis refer to the molecular weight of the amplification products, whereas coordinates on Y-axis refer to the intensity of the PCR products. Common alleles of samples in the same column are highlighted in grey and the allele size is given in the upper part (TIF 109 KB)

Supplementary Table 1: The SSR genotypes and basic statistics of 17 polyploid *Prunus* accessions analysed in nine loci (XLS 93 KB)

Supplementary Table 2: The self-incompatibility ribonuclease (*S-RNase*) alleles sequenced in the present study (DOCX 15 KB)

Supplementary Table 3: Estimates of evolutionary divergence between pairs of amino acid sequences of polyploid *Prunus S-RNase* alleles calculated using the Poisson correction model in MEGA 5.1 (XLS 33 KB)
