# Supplementary figures and images for: Simple Sequence Repeat and S-Locus Genotyping to Assist the Genetic Characterization and Breeding of Polyploid Prunus Species, P. spinosa and P. domestica subsp. insititia
Source: Biochem Genet. 2021 Jun 16;59(4):1065–87. doi: 10.1007/s10528-021-10090-7 (PMC8249305; doi:10.1007/s10528-021-10090-7)

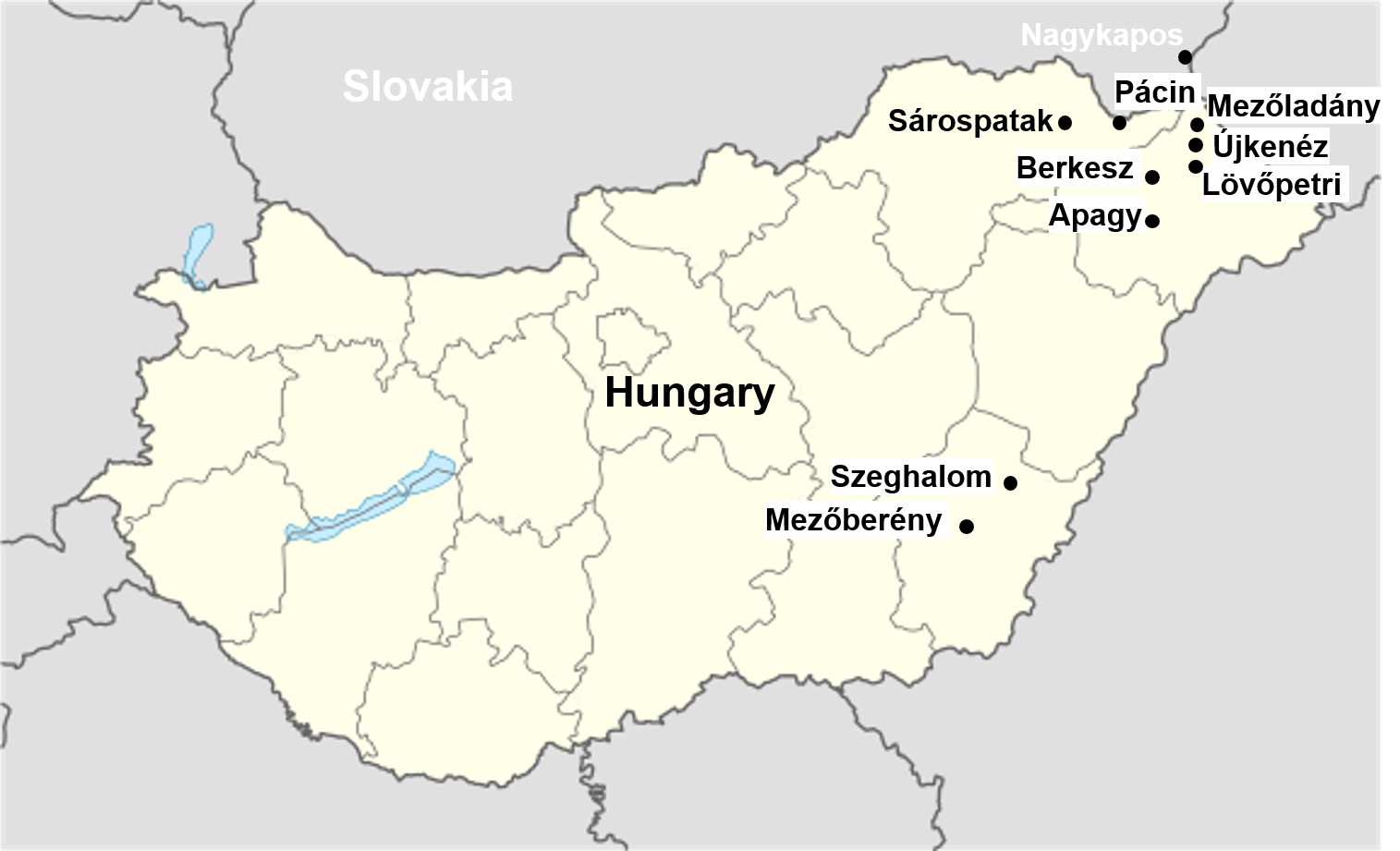

Supplement: Supplementary file 1 — Supplementary Figure 1: The geographic origin of the analysed Central European Prunus spinosa, P. domestica subsp. insititia and P. spinosa × P. domestica accessions (TIF 536 KB) [file 10528_2021_10090_MOESM1_ESM.tif]

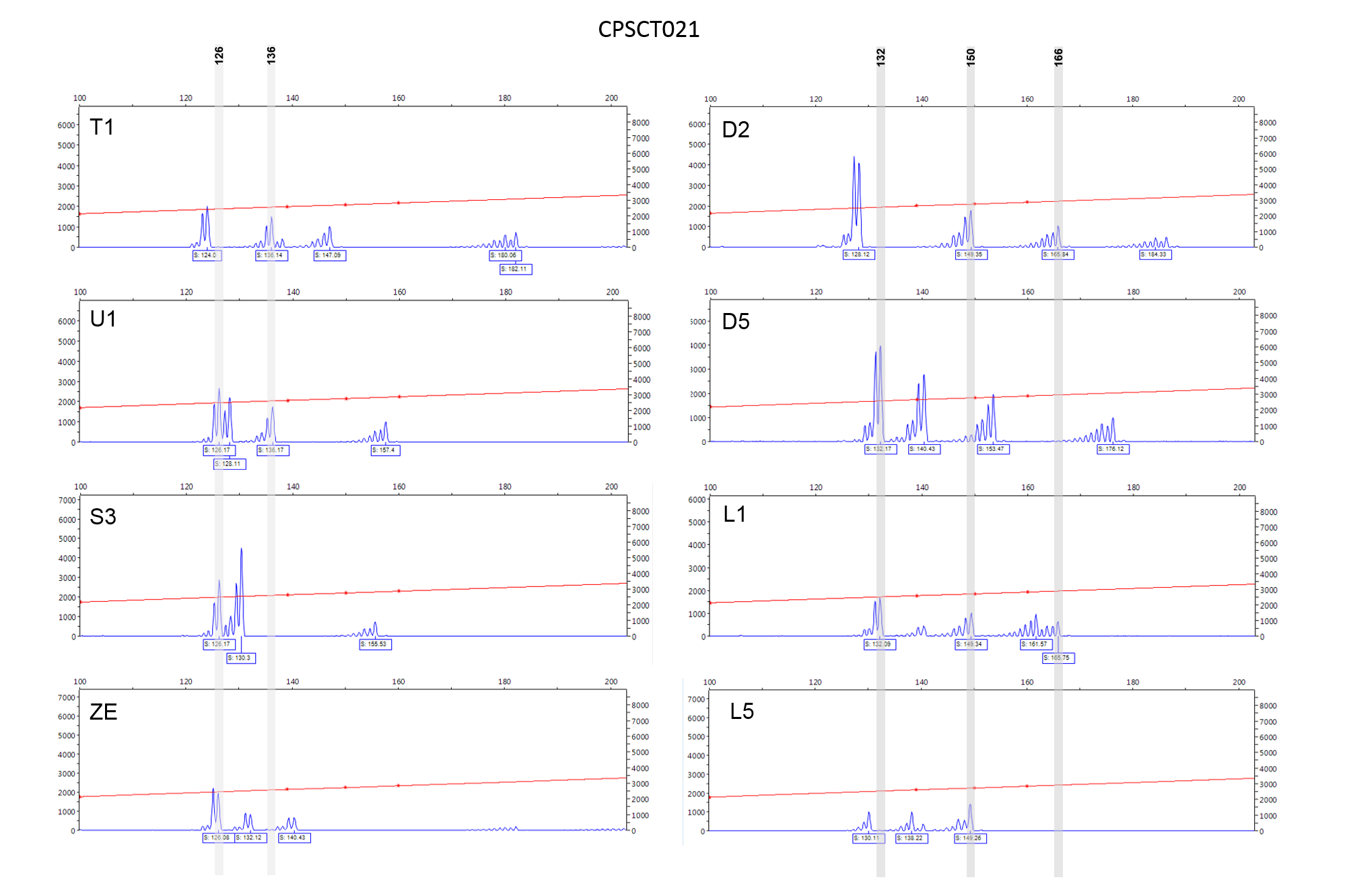

Supplement: Supplementary file 2 — Supplementary Figure 2: Unweighted pair-group average (UPGMA) dendrogram based on Dice indices among SSR in 9 loci and S-genotypes of 17 native Prunus spinosa (squares), P. domestica subsp. insititia (asterisks) and P. spinosa × P. domestica hybrid (hexagons) accessions. Numbers indicate bootstrap values (percentage of 2000 replicates). Bootstrap values greater than 50% are shown (TIF 638 KB) [file 10528_2021_10090_MOESM2_ESM.tif]

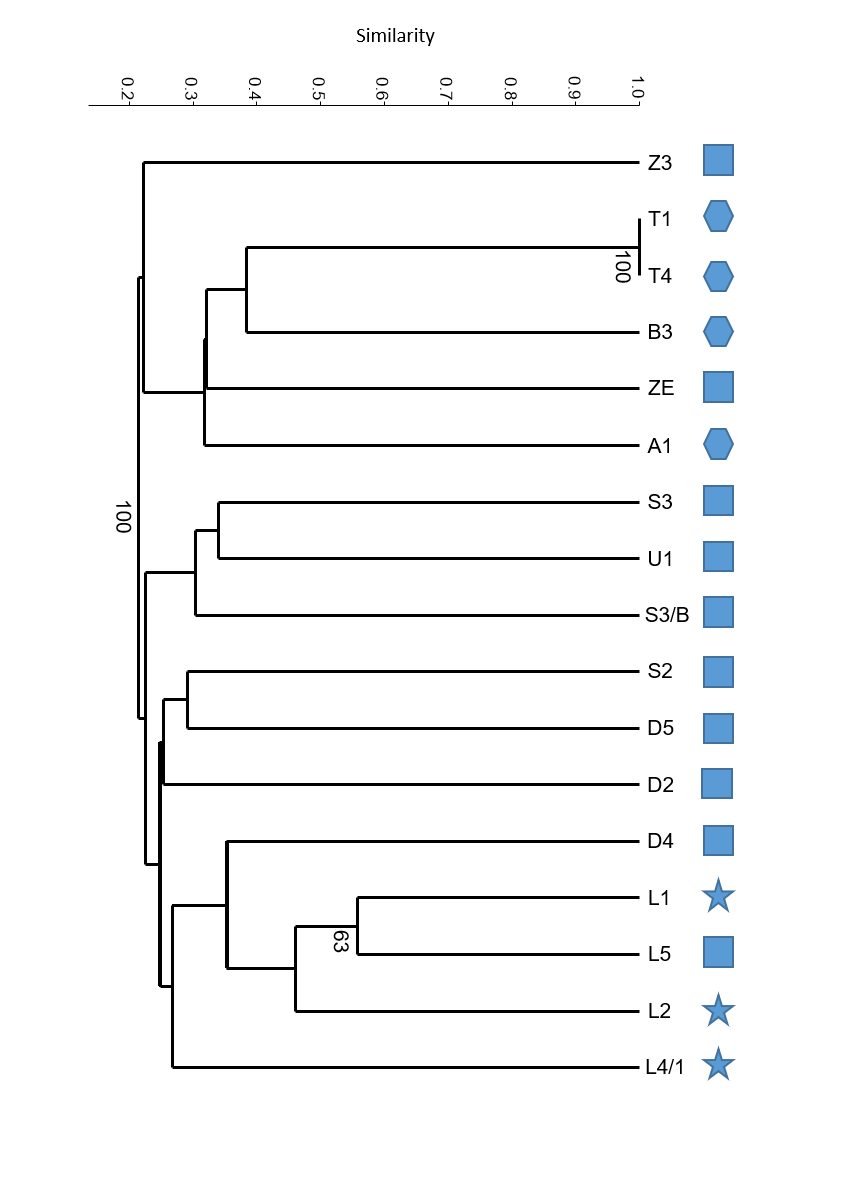

Supplement: Supplementary file 3 — Supplementary Figure 3: Representative Peak Scanner traces for the CPSCT021 microsatellite alleles amplified with 6-FAM-labelled primers in ten polyploid Prunus accessions and GS500 LIZ size standard. Coordinates on the X-axis refer to the molecular weight of the amplification products, whereas coordinates on Y-axis refer to the intensity of the PCR products. Common alleles of samples in the same column are highlighted in grey and the allele size is given in the upper part (TIF 109 KB) [file 10528_2021_10090_MOESM3_ESM.tif]
