# Supplementary material for: Simple Sequence Repeat and S-Locus Genotyping to Assist the Genetic Characterization and Breeding of Polyploid Prunus Species, P. spinosa and P. domestica subsp. insititia
Source: Biochem Genet. 2021 Jun 16;59(4):1065–87. doi: 10.1007/s10528-021-10090-7 (PMC8249305; doi:10.1007/s10528-021-10090-7)
Supplement: Supplementary file 5 — Supplementary Table 2: The self-incompatibility ribonuclease (S-RNase) alleles sequenced in the present study (DOCX 15 KB) [file 10528_2021_10090_MOESM5_ESM.docx]

| *S-RNase* allele | Genotype | Accession number | The most similar sequence | Percentage identity | E-value |
| --- | --- | --- | --- | --- | --- |
| *S*_3-1_ | T1 | MN052897 | *P. spinosa* *S*_3-1_ | 99.6 | 0.0 |
|  | D5 | MN052898 | *P. spinosa* *S*_3-1_ | 99.2 | 0.0 |
| *S*_12_ | D4 | MN052899 | *P. spinosa S*_12_ | 98.1 | 0.0 |
| *S*_A_ | A1 | MN069629 | *P. armeniaca S*_17_ | 98.3 | 0.0 |
| *S*_B_ | D2 | MN069630 | *P. virginiana S*_4_ | 97.8 | 2e-174 |
|  | L1 | MN069631 | *P. virginiana S*_4_ | 97.8 | 2e-174 |
|  | Z3 | MN069632 | *P. virginiana S*_4_ | 98.3 | 3e-173 |
| *S*_C_ | B3 | MN069633 | *P. humilis S*_10_ | 98.4 | 0.0 |
|  | L1 | MN069634 | *P. humilis S*_10_ | 97.8 | 1e-173 |
| *S*_D_ | T1 | MN069635 | *P. speciosa* *S*_37_ | 86.8 | 5e-172 |
|  | Z3 | MN069636 | *P. speciosa* *S*_37_ | 86.6 | 2e-170 |
| *S*_J_ | B3 | MN069637 | *P. pseudocerasus S*_4_ | 93.0 | 0.0 |
| *S*_K_ | D4 | MN069638 | *P. armeniaca S*_29_ | 89.7 | 0.0 |
| *S*_M_ | D2 | MN069639 | *P. mume S*_4_ | 95.8 | 0.0 |
|  | L1 | MN069640 | *P. mume S*_4_ | 95.8 | 0.0 |
| *S*_P_ | D5 | MN069641 | *P. cerasifera S*_9_ | 92.6 | 0.0 |
| *S*_U_ | T1 | MN069642 | *P. dulcis S*_52_ | 96.4 | 0.0 |
